# Outcomes in 1096 patients with severe thrombotic thrombocytopenic purpura before the Caplacizumab era

**DOI:** 10.1371/journal.pone.0256024

**Published:** 2021-08-12

**Authors:** Andry Van de Louw, Eric Mariotte, Michael Darmon, Austin Cohrs, Douglas Leslie, Elie Azoulay

**Affiliations:** 1 Division of Pulmonary and Critical Care Medicine, Pennsylvania State University College of Medicine and Milton S. Hershey Medical Center, Hershey, PA, United States of America; 2 Intensive Care Department, University of Paris, Saint-Louis Hospital, Paris, France; 3 Department of Public Health Sciences, Pennsylvania State University College of Medicine and Milton S. Hershey Medical Center, Hershey, PA, United States of America; Universite de Liege (B34), BELGIUM

## Abstract

**Introduction:**

Thrombotic thrombocytopenic purpura (TTP) is a diagnostic and therapeutic emergency. Therapeutic plasma exchange (TPE) combined with immunosuppression has been the cornerstone of the initial management. To produce optimal benefits, emerging treatments must be used against a background of best standard of care. Clarifying current uncertainties is therefore crucial.

**Methods:**

The objective of this study was to analyze a large high-quality database (Marketscan) of TTP patients managed between 2005 and 2014, in the pre-caplacizumab era, in order to assess the impact of time to first TPE and use of first-line rituximab on mortality, and whether mortality declines over time.

**Results:**

Among the 1096 included patients (median age 46 [IQR 35–55], 70% female), 28.8% received TPE before day 2 in the ICU. Hospital mortality was 7.6% (83 deaths). Mortality was independently associated with older age (hazard ratio [HR], 1.024/year; 95% confidence interval [95%CI], [1.009–1.040]), diagnosis of sepsis (HR, 2.360; 95%CI [1.552–3.588]), and the need for mechanical ventilation (HR, 4.103; 95%CI, [2.749–6.126]). Factors independently associated with lower mortality were TPE at ICU admission (HR, 0.284; 95%CI, [0.112–0.717]), TPE within one day after ICU admission (HR, 0.449; 95%CI, [0.275–0.907]), and early rituximab therapy (HR, 0.229; 95% CI, [0.111–0.471]). Delayed TPE was associated with significantly higher costs.

**Conclusions:**

Immediate TPE and early rituximab are associated with improved survival in TTP patients. Improved treatments have led to a decline in mortality over time, and alternate outcome variables such as the use of hospital resources or longer term outcomes therefore need to be considered.

## Introduction

Patients with severe thrombotic thrombocytopenic purpura (TTP) are at high risk of ischemic organ damage [1]. The disease is due to a deficiency in the serine metalloprotease ADAMTS13 that specifically cleaves von Willebrand factor (VWF). The VWF therefore remains in the form of ultra-large multimers that bind to platelets in high-shear environments, resulting in microthrombi and subsequent microvascular occlusion [2–4]. TTP is a diagnostic and therapeutic emergency [5, 6].

Over the last two decades, advances in the understanding of the pathophysiology of TTP [7, 8] and efforts to standardize the initial management [9–11] have significantly decreased mortality in patients with TTP. The most important improvement is the routine use of plasma exchange [12]. Although no randomized controlled trials are available, convincing data exist [13–15] to support a role for steroids, rituximab, and other immunomodulatory therapies, which have become front-line treatments for TTP [11]. A phase 2 and a phase 3 randomized controlled trial demonstrated that caplacizumab, an anti-VWF nanobody that inhibits the interaction between VWF and platelets, when used in combination with plasma exchange and immunosuppression, resulted in a significantly shorter time to the platelet count response compared to plasma exchange and immunosuppression alone [16, 17]. Caplacizumab was approved in 2019, and post-marketing data are not yet available.

Several unanswered questions remain about patients with severe TTP in the pre-caplacizumab era [5]. For instance, performing the first plasma exchange as early as possible has been recommended, as without this treatment patients are at risk for fatal cardiovascular events [10, 18]. However, no study has properly evaluated the association between time to first plasma exchange and mortality. Second, rituximab has been recommended as a first-line treatment to improve the short-term response to plasma exchange, expedite platelet recovery [14], reduce the relapse rate, and improve longer term outcomes [19–22]. Nevertheless, whether early rituximab administration, as recommended by experts [5, 10], has beneficial effects on outcomes such as TTP remission, early relapse rate, and mortality remains unclear. Last, several studies published over the last 20 years suggest that survival rates have improved over time. However, mortality rates have not been compared across different time periods in a large longitudinal study that included patients throughout an entire decade.

To address these three questions, we analyzed a large high-quality database that included 1096 patients with TTP over a 10-year period.

## Materials and methods

This retrospective cohort study used 2005–2014 data from the IBM MarketScan database to describe the characteristics of patients diagnosed with thrombotic microangiopathy (TMA) and treated with therapeutic plasma exchange (TPE). The Penn State Health institutional review board approved the study and waived the requirement for informed consent, given the retrospective design. The database is a commercially available health insurance claims database. It includes claims data for more than 245 million privately insured people in all 50 US states, including demographic characteristics, healthcare utilization and costs, dates of service, diagnosis codes, and procedure codes. For the years 2005–2014, ICD-9 was the classification system used in the database. The data represent claims from clinicians, hospitals, and pharmacies that have been adjudicated for payment and are obtained directly from a convenience sample of large employers and health plans that agree to participate in the database. The data used in this study were entered in the database between 2005 and 2015 as claims were adjudicated and information sent to Marketscan by participating health insurances. MarketScan does not include patients on Medicare (≥ 65 years of age). IBM Marketscan has a quality-control process to verify that the data meet criteria for quality and completeness [23]. This database has been used in multiple other studies [23–25] including studies examining complications and follow-up care after healthcare procedures [23].

We included all adults (>18 years of age) with hospital inpatient admissions associated with a diagnosis code of TMA (ICD-9 446.6) and a procedure code of TPE (ICD-9 PCS 997.1 or CPT 36514, 36515 OR 36516) between 2005 and 2014. Patients with a diagnosis of hemolytic and uremic syndrome (ICD-9 283.11) were excluded, so that included patients were believed to have undergone TPE for TTP.

### Data collected

The database was accessed multiple times by the data manager (AC) between June 2018 and June 2019 to collect the data used in this study. First inpatient admissions for the study population were screened, and data reported in sps and figures were collected. Geographic location (State), ICD-9 principal and secondary diagnosis (up to 15 per admission) and procedure codes (up to 15 per admission), total cost of admission, and discharge status were among the collected variables. Dates of TPE procedures and rituximab administration (to inpatients or outpatients) were recorded. To assess survival over time, we used discharge status for the last inpatient admission (regardless of diagnoses or procedures), physician office visits, outpatient prescription fillings, and disenrollment from the database (whichever occurred latest). Inpatient admission cost was collected: it included insurance payment and any patient`s out-of-pocket payment and covered all services (physician fees, medications administered, tests, etc.) provided during inpatient admission.

Time to first TPE was defined as the time elapsed between the day of admission and the day of the first TPE for the first inpatient admission. The ICD-9 codes 584.9 (acute kidney failure, unspecified), 586 (renal failure, unspecified) and 584.5 (acute kidney failure with tubular necrosis) were used to define acute kidney injury and codes corresponding to cerebral infarction or transient cerebral ischemia (434.91, 435.9, 436, 784.3), convulsions (780.9, 345.9), and encephalopathy or psychosis (780.97, 348.3, 348.39, 780.09, 298.9) were used to define severe neurological complications. Corresponding ICD-9 codes were also screened for acute myocardial infarction (410.X), cardiogenic shock (785.51), and cardiac arrest (427.5). Likewise, CPT or HCPCS codes were used to collect corresponding procedures.

As a surrogate for TTP relapses, we used subsequent admissions with TPE performed more than 30 days after discharge from the first admission.

### Statistical analysis

Quantitative variables are described as median (interquartile range [IQR]) and are compared between groups using the non-parametric Wilcoxon rank-sum test. Qualitative variables are described as frequency (percentages) and are compared between groups using Fisher’s exact test. Mortality was assessed using survival analysis at a horizon of 3 years.

Independent risk factors for mortality were assessed using a Cox model. Conditional stepwise variable selection was performed with 0.2 as the critical *P* value for entry into the model and 0.1 as the *P* value for removal. Interactions and correlations between the explanatory variables were carefully checked. The validity of the proportional hazard assumption, influence of outliers, and linearity of the relationship between the log hazard and the covariates were carefully checked. The preplanned statistical analysis stated that the final model used would be the selected Cox model along with admission state and year as frailty terms. Before the analysis, we also planned to force year and time from admission to TPE initiation into the model should these variables not be selected.

Independent risk factors for costs were assessed using mixed linear regression, reporting results as US $. Conditional stepwise variable selection was performed with 0.2 as the critical *P* value for entry into the model and 0.1 as the *P* value for removal. Interactions and correlations between the explanatory variables were carefully checked. Linearity, homoscedasticity, independence, and normality assumptions were checked. The statistical plan stated that the final model used would be the selected linear model with state as a random effect on the intercept and year as a random effect on the slope [26].

Kaplan-Meier graphs were drawn to express the probability of death from inclusion to 3 years. Comparisons were performed using the log-rank test. Statistical analyses were performed with R statistical software, version 3.4.3 (available online at http://www.r-project.org/), using the packages ‘Survival’, ‘coxme’,’lme4’,’ and ‘lmerTest’. *P* values <0.05 were considered significant.

## Results

**Fig 1:**
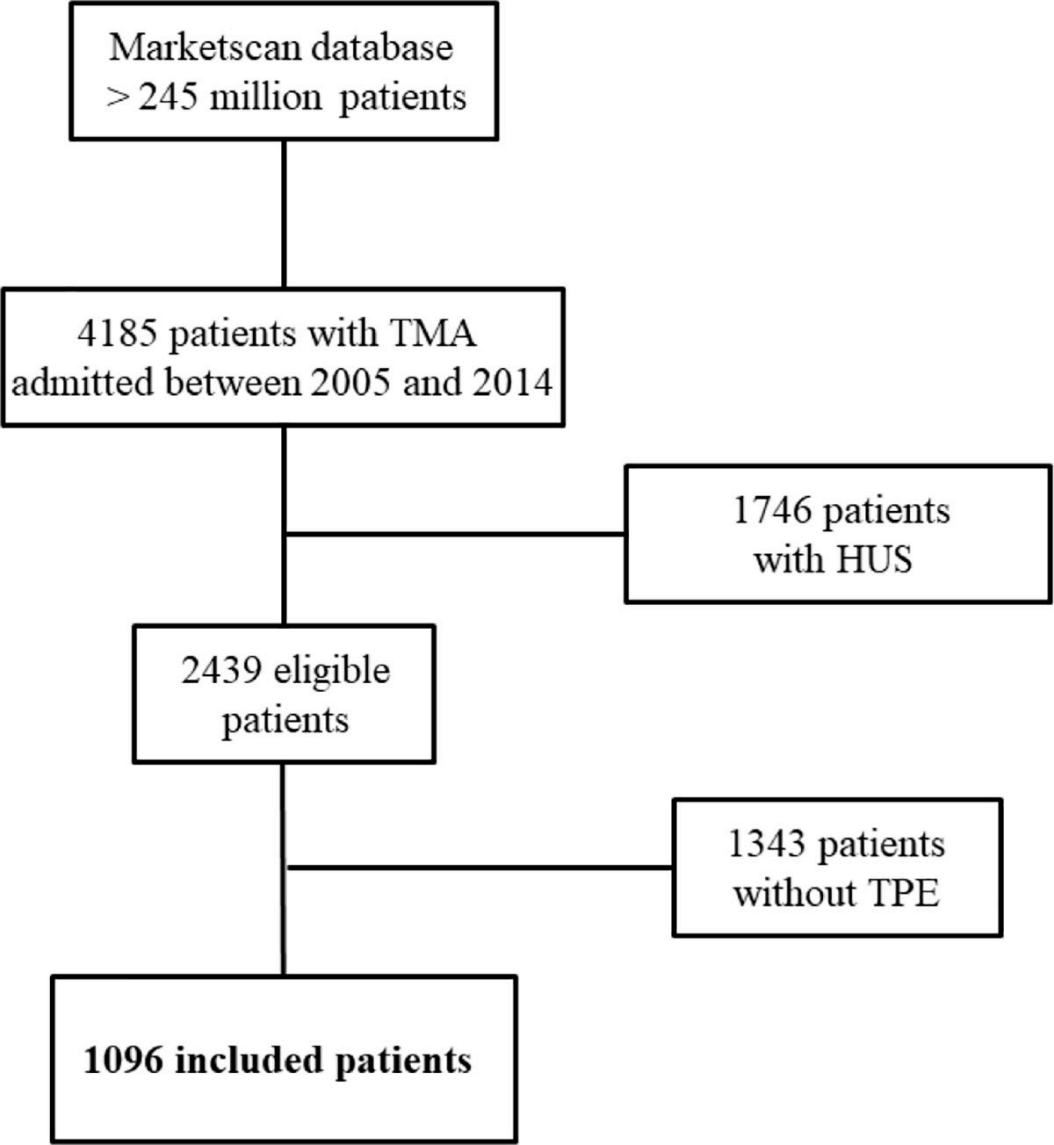
Study flowchart.

We included 1096 patients (Fig 1 is the flowchart). Table 1 reports the patients’ characteristics. Median age was 46 y [35–55] overall and 70% of patients were females. Significant comorbid conditions included chronic kidney disease (n = 127, 11.6%), autoimmune disease (n = 82, 7.5%), and HIV infection (n = 42, 3.8%).

**Table 1:** Patients’ characteristics and diagnoses associated with TTP.

N (%) or Median (IQR)	Alive (n = 964)	Deceased (n = 132)	*P* value
**Age, years, median [IQR]**	45 [35–54]	52 [42–58]	<0.001
**Females, n (%)**	683 (70.9)	86 (65.2)	0.215
**Year of admission, n (%)**			0.23
**2005–2008**	321 (33.3)	53 (40.2)	
**2009–2010**	202 (21.0)	31 (23.5)	
**2011–2012**	247 (25.6)	26 (19.7)	
**2013–2014**	194 (20.1)	22 (16.7)	
**Acute kidney injury, n (%)**	376 (39.0)	72 (54.5)	0.001
**Hypertension, n (%)**	264 (27.4)	50 (37.9)	0.016
**Neurological signs, n (%)**	68 (7.1)	9 (6.8)	1
**Sepsis, n (%)**	70 (7.3)	37 (28.0)	<0.001
**Cardiogenic shock, n (%)**	5 (0.5)	2 (1.5)	0.18
**Acute myocardial infarction, n (%)**	44 (4.6)	12 (9.1)	0.18
**Cardiac arrest, n (%)**	4 (0.4)	10 (7.6)	<0.001

Acute kidney injury was reported in 448 (41%) patients, of whom 175 (16%) required renal replacement therapy. Hypertension was noted in 289 (26%) patients. Neurological signs, found in 314 (28.6%) patients, included headaches (n = 135, 12%), seizures (n = 77, 7%), stroke (n = 92, 8%), and transient cerebral ischemia (n = 52, 5%). Severe cardiac involvement included signs suggestive of acute myocardial infarction (n = 56, 5%) and cardiogenic shock (n = 7, 0.6%). A clinical diagnosis of sepsis was established in 107 (9.8%) patients, including 58 who were hypotensive at ICU admission. Vital organ support (vasopressors, mechanical ventilation, and/or renal replacement therapy) was unnecessary in 80% of patients, whereas 17% of patients required support of one vital organ and 3% of two vital organs. Cardiac arrest occurred in 14 (1%) patients.

Table 2 shows the main therapeutic interventions. Only 316 (28.8%) patients received TPE at or within 1 day after ICU admission. However, time to first TPE was unknown for two-fifths of patients. At some point during the ICU stay, renal replacement therapy was required for 175 (16%) patients, mechanical ventilation for 99 (9%) patients, vasopressors for 7 (0.6%) patients, and extracorporeal membrane oxygenation for 7 (0.6%) patients.

**Table 2:** Specific investigations, therapeutic interventions, and outcomes.

N (%) or Median (IQR)	Alive (n = 964)	Deceased (n = 132)	*P* value
**Investigations, n (%)**			
***Brain MRI***	188 (19.5)	35 (26.5)	0.078
***Head CT***	323 (33.5)	64 (48.5)	0.001
***Echocardiography***	302 (31.3)	66 (50)	<0.001
**Therapeutic interventions, n (%)**			
***Time to first plasma exchange***			<0.001
*** On the day of admission***	128 (13.3)	5 (3.8)	
*** One day***	169 (17.5)	14 (10.6)	
*** Two days***	71 (7.4)	9 (6.8)	
*** >2 days***	183 (19.0)	54 (40.9)	
*** Unknown***	413 (42.8)	50 (37.9)	
***TPE frequency***			0.03
*** Daily***	374 (38.8)	48 (36.4)	
*** Alternate days***	56 (5.8)	7 (5.3)	
*** 72 h or more***	49 (5.1)	6 (4.5)	
*** Unknown***	413 (42.8)	50 (37.9)	
***Number of TPE sessions*** ^***a***^	5 (3–8)	4 (1.25–9)	0.17
***Rituximab administration***	260 (27.0)	8 (6.1)	<0.001
***Mechanical ventilation***	54 (5.6)	45 (34.1)	<0.001
***Renal replacement therapy***	138 (14.3)	37 (28.0)	<0.001
***Vasopressors***	3 (0.3)	4 (3.0)	0.002
***ECMO***	2 (0.2)	5 (3.8)	<0.001
***Red blood cell transfusion***	199 (20.6)	27 (20.5)	0.96
***Plasma transfusion***	171 (17.7)	19 (14.4)	0.34
***Platelet transfusion***	57 (5.9)	12 (9.1)	0.16
**Outcomes, median [IQR]**	39,190	89,502	<0.001
***Inpatient admission cost (USD)***	[18 147–99 13]	[31 430–205 929]	
***Hospital LOS (days)***	11 [7–19]	16.5 [7–32.5]	0.002

^a^ available for 633 patients.

MRI, magnetic resonance imaging; CT, computed tomography; TPE, therapeutic plasma exchange; ECMO, extracorporeal membrane oxygenation; USD, US dollars; LOS, length of stay

Of the 1096 patients, 83 (8%) died, within 13 (7–34) days of admission. Median follow-up after hospital discharge in survivors was 547 days [130–1169]. Median hospital length of stay was 11 (7–20) days. Discharge status was available for 962 survivors and was distributed as follows: home (n = 773, 73%), home with healthcare service (n = 80, 8%), short-term hospital (n = 47, 4%), inpatient rehabilitation (n = 22, 2%), long-term facility (n = 23, 2%), hospice (n = 12, 1%), or left against medical advice (n = 5, 0.5%). Relapses occurred in 103 (9%) patients.

By multivariable analysis (Table 3), mortality was independently and positively associated with older age, diagnosis of sepsis, and the need for mechanical ventilation. Protective factors were first TPE at ICU admission or within one day after ICU admission and rituximab administration.

**Table 3:** Results of the Cox mixed-effects model analyzing mortality in patients with thrombotic thrombocytopenic purpura treated with therapeutic plasma exchange.

Variable	Hazard ratio	95% confidence interval	*P* value
**Age**	1.024/year	1.009–1.040	0.002
**First TPE after day 2**	Ref	-	-
**First TPE at ICU admission**	0.284	0.112–0.717	0.008
**First TPE on day 1**	0.449	0.275–0.907	0.02
**First TPE on day 2**	0.776	0.377–1.598	0.49
**First TPE at unknown time**	0.742	0.496–1.112	0.15
**Mechanical ventilation**	4.103	2.749–6.126	<0.0001
**Vasopressors**	2.624	0.944–7.290	0.064
**Sepsis**	2.360	1.552–3.588	<0.0001
**Renal replacement therapy**	0.928	0.606–1.421	0.73
**Rituximab administration**	0.229	0.111–0.471	<0.0001

TPE, therapeutic plasma exchange

Sensitivity analyses were performed based on platelet transfusion, presence of auto-immune disease, and number of diagnostic tests performed (head CT, MRI), as well as an analysis adjusted on number of vital organ dysfunctions. The results were similar to those of the main analysis. For patients who received rituximab, the first dose was administered 34 (20–88) days after admission, and a sensitivity analysis restricted to patients with early (within 34 days, median value) administration showed similar protective effect of rituximab on mortality (Hazard Ratio 0.07, 95% confidence interval 0.01–0.57, p = 0.01).

**Fig 2:**
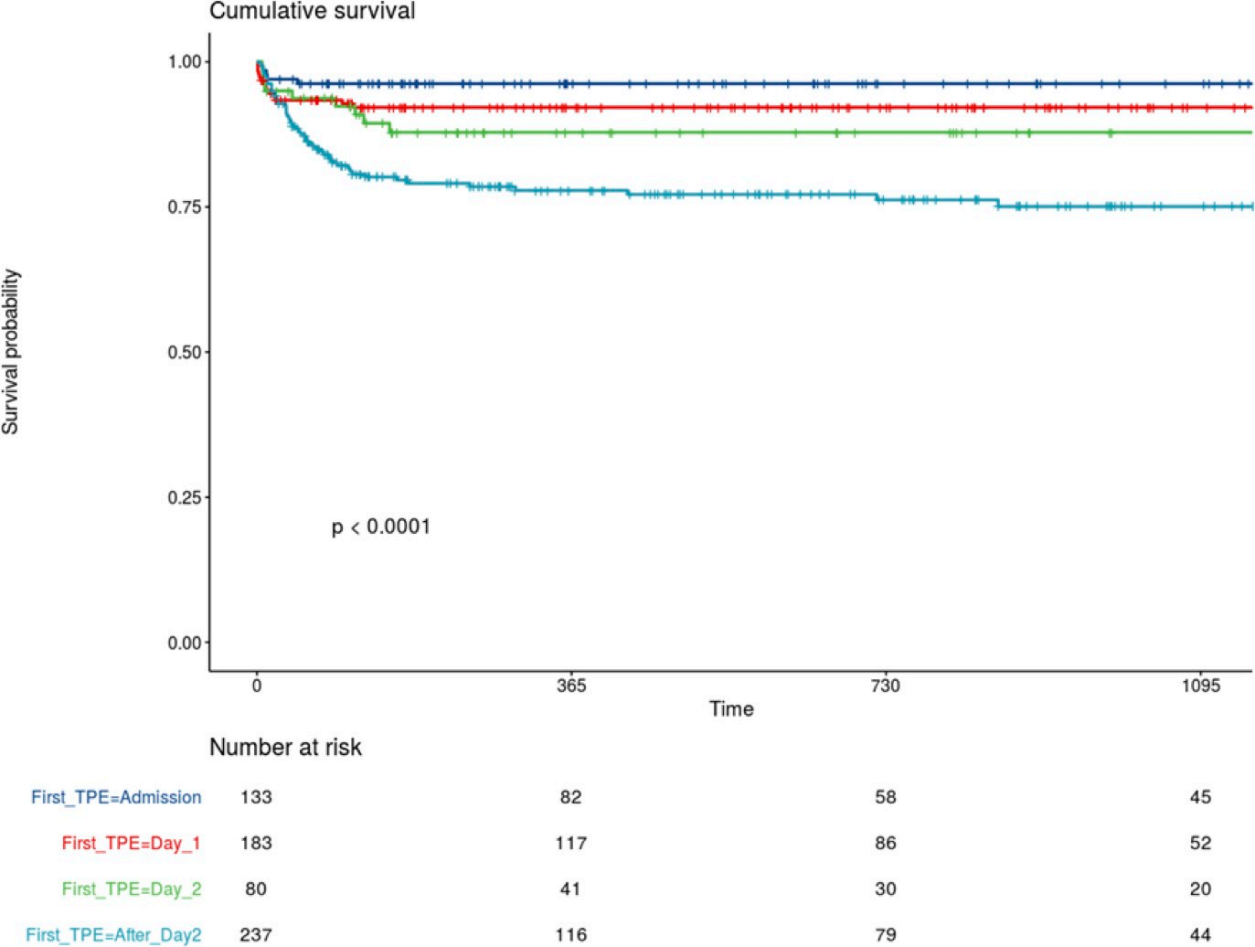
Survival analysis. Kaplan-Meier curves of survival probability for patients with TTP grouped by timing of first TPE (day of admission, day 1, day 2, after day 2). Logrank test showed a significant effect of TPE timing on long-term survival (*P*<0.0001).

**Fig 3:**
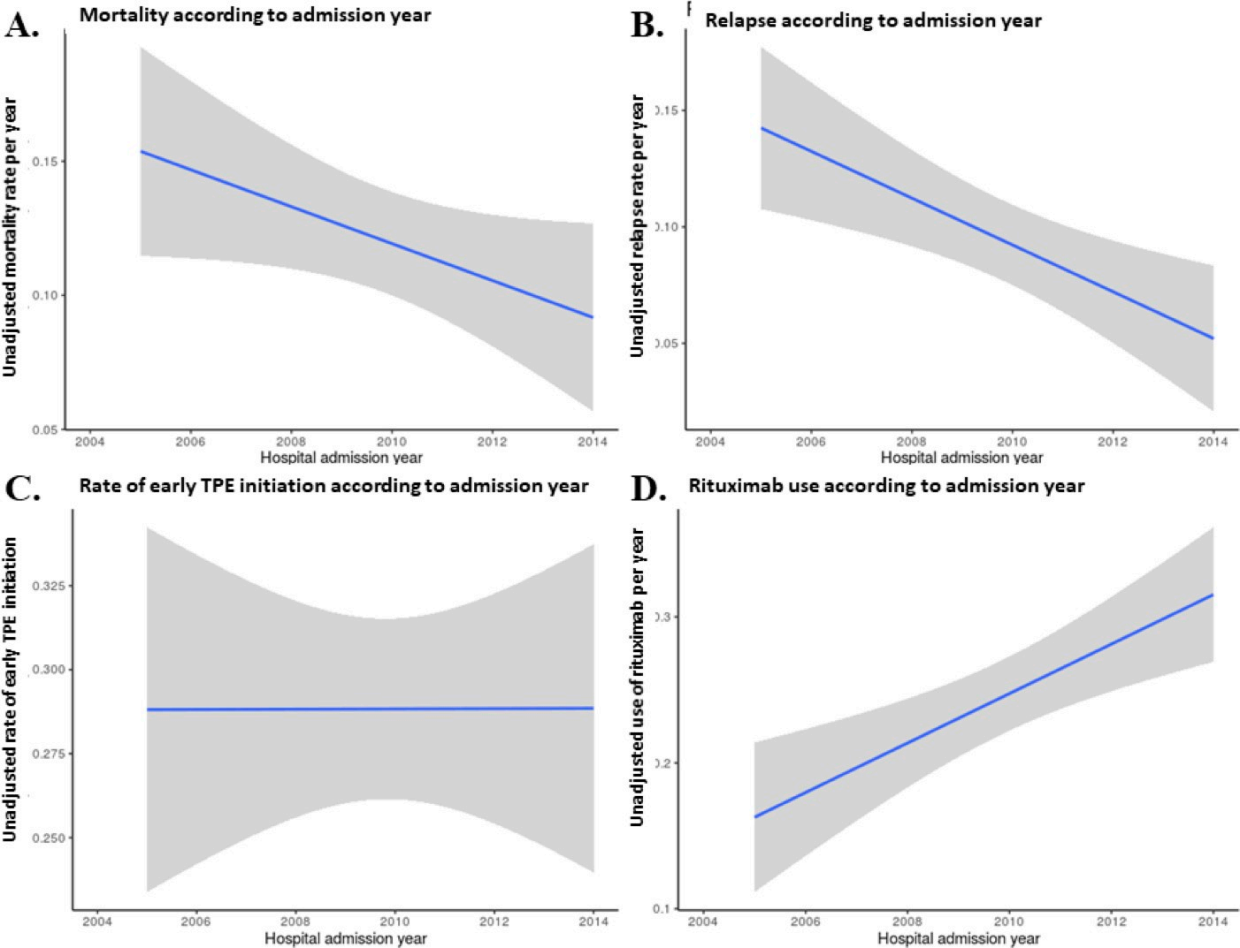
Trends of mortality, relapse, early TPE initiation and rituximab use over time. Unadjusted rates of mortality (**A**), relapse (**B**), early TPE initiation, (**C**) and rituximab use (**D**) in patients with TTP according to the year of admission. Gray areas reflect 95% confidence interval of changes.

Fig 2 displays Kaplan Meier survival curves according to time to first TPE: the shorter the time to first TPE, the better survival (*P*<0.0001). As shown in Fig 3, mortality and relapse rates diminished significantly over the years.

The total cost associated with TTP admissions was 42 593 [18 904–110 424] USD. Sepsis, mechanical ventilation, and delayed TPE (after day 2) were associated with higher costs (Table 4).

**Table 4:** Results of the linear mixed-effects model analyzing cost (in USD) in patients with thrombotic thrombocytopenic purpura treated with therapeutic plasma exchange.

Variable	Estimate	95% confidence interval	*P* value
**Intercept**	66 448	41,637–91,260	<0.0001
**Mechanical ventilation**	82 990	53,411–112,569	<0.0001
**Epilepsy**	29 142	-3,018–61,302	0.08
**Sepsis**	78 622	50,397–106,848	<0.0001
**First TPE on day 1**	-17 421	-48,119–13,276	0.27
**First TPE on day 2**	-4465	-42,513–33,582	0.82
**First TPE after day 2**	84 823	55,379–114,266	<0.0001
**First TPE at unknown time**	-20 674	-47,439–6,091	0.13

TPE: therapeutic plasma exchange

## Discussion

Advances in the understanding and treatment of TTP have not only translated into survival benefits [12] but also decreased organ dysfunctions related to microvascular injury [27], shortened time to remission, and decreased the risk of relapses [9]. The recent release of caplacizumab has committed clinicians to better define the standard of care to which this drug should be added [5]. Caplacizumab compared with placebo significantly reduced the time to platelet normalization, exacerbations, and relapses [16, 17]. Our study on TTP patients not treated with caplacizumab showed that early TPE was associated with survival benefits and reduced costs and that the early use of rituximab was associated with improved survival. Therefore, early TPE and rituximab should be part of the standard of care for TPE, which forms the background against which any emerging treatments should be evaluated. Moreover, by showing that mortality declined over the years even during a single decade, this study indicates that studies of TTP-related mortality involving comparisons with historical controls should be cautiously interpreted. This decline in mortality over recent decades has also been reported in other ICU populations, like patients with sepsis [28], and could be partly due to improvement in quality of care in general.

TPE is the first treatment proven effective in patients with TTP [12]. TPE produced better survival rates compared to either no plasma therapy or plasma infusion. For years, TPE has been the only life-saving treatment for TTP. The issue of the timing of TPE, however, had not been well investigated. Many studies evaluated outcomes in patients with or without TPE but did not separate patients into groups depending on TPE timing [12, 27, 29, 30]. In one study, among patients who died from acute TTP, a large proportion died before having received TPE [18]. Our study adds to the existing knowledge by demonstrating that TTP should be given at ICU admission or, at the latest, on the first ICU day.

No randomized controlled trial has properly demonstrated that rituximab provided benefits to patients with TTP receiving TPE [31, 32]. However, data suggest that rituximab improves short and long term clinical outcomes. For instance, two studies compared rituximab-treated patients to historical controls. When rituximab therapy was added to the treatment of 22 patients with no response to TPE, a significant reduction in the time to a durable remission was achieved, with higher ADAMTS 13 activity and lower ADAMTS 13 antibody titers, compared to historical controls [21]. Another study assessed rituximab started within 3 days of acute TTP admission in addition to TPE and steroids [14]. Compared to historical controls, rituximab-treated patients had a shorter length of stay and significantly reduced relapse rate [14]. We similarly observed that, as rituximab use increased over time in our series, relapse rate decreased. Our study is the first to suggest that early rituximab use is also associated with lower mortality.

In the Oklahoma TTP registry, approximately 50% of the 78 patients with TTP included between 1995 and 2015 had some degree of acute kidney injury and only 4% required hemodialysis [33]. By contrast in another series of 92 patients, all admitted to the ICU of a referral tertiary care center with documented TTP, 59% had acute kidney injury and 15% required renal replacement therapy [34]. Selection of patients with different severity of illness may account for these differences; our results are similar to the latter study (16% of patients required renal replacement therapy), suggesting that our patients had severe TTP.

The incidence of neurological signs in our series (about 7%) is lower than reported in most previously published studies (39%-80% including minor symptoms in a recent review [35]) and likely underestimated. As we used ICD-9 diagnoses to define neurological signs, it is probable that underreporting, especially of minor neurological symptoms, accounts for this discrepancy.

The number of TPE sessions performed in our patients (median = 5) appears low but should be interpreted with caution as information was missing for approximately 40% of the population. Median numbers of TPE sessions required to achieve remission vary widely in the literature, some studies reporting median of 4–7 sessions [36–39] while others reported numbers above 10 [40, 41]. Patient selection, severity of illness and associated therapies [42] might account for this large range. This should be taken into account when comparing pre- and post-Caplacizumab era data. In the recent HERCULES trial [43], the median number of days of TPE was 7 in the placebo group versus 5 in the Caplacizumab group.

This study has several limitations. First, the ADAMTS 13 concentration–the biological signature of TTP–was not available in the database, raising concerns that some of the included patients might not have had genuine TTP. However, in this highly reliable database, all patients had thrombotic microangiopathy, patients with hemolytic and uremic syndromes were excluded, and only patients receiving TPE were included. The combination of these criteria clearly points towards TTP as the etiology for TMA and the same approach has been used in previous studies to define patients with TTP [44]. Moreover, the proportions of patients with organ dysfunction and study outcomes were in the same ranges as in previously published TTP studies [8, 14, 18, 45, 46]. Second, while TTP treatment includes TPE and immunomodulatory drugs, data on the use of steroids were not available in our study, raising concern that first-line rituximab might have been used instead of steroids. However, it is likely that the vast majority of patients received steroids, in agreement with available guidelines and practices [10, 33, 42, 47]. Although recommended [5, 9, 48, 49] the level of evidence for the use of steroids in patients with TTP is far less strong than that of rituximab. For instance, no proper randomized controlled trial has compared clinical outcomes in TTP patients treated with vs. without steroids, in addition to the standard of care. An early study evaluated a protocol of first-line steroids alone (200 mg of prednisone/day) in patients with thrombotic microangiopathy related to TTP or HUS with minimal symptoms and no central nervous system symptoms. TPE was provided only in case of rapid clinical deterioration, unresponsiveness after 48 hours of prednisone alone, or rapidly declining hematocrit values and platelet counts [50]. Prednisone alone was effective in 28% (30/108) of patients with mild TTP-HUS. The other study that argues for the use of steroids in patients with TTP randomized patients to either standard (1mg/kg/day) or high-dose (10 mg/kg/day for 3 days) methylprednisolone as an adjunctive treatment to TPE in patients with TTP [13]. The proportion of patients achieving remission was significantly higher in the high-dose methylprednisolone group compared to the standard methylprednisolone group (23% vs. 53%). Third, most patients in our study were admitted to the intensive care unit, suggesting that our results may apply only to patients with severe TTP. However, only a minority of patients needed life-sustaining interventions. The ICU is probably the most appropriate place for the initial management of patients with TTP who require urgent TPE and may require invasive procedures despite deep thrombocytopenia [5]. Fourth, the timing of TPE in our study may be a surrogate for how quickly the diagnosis of TTP was established and our results may therefore highlight the importance of a rapid diagnosis (and quick initiation of steroid therapy for instance) rather than rapid TPE initiation. Likewise, patients who underwent late TPE may represent a subset with ‘atypical’ TTP (associated with cancer or transplant for instance) for whom the use of TPE was more questionable. Finally, in our study including patients at the pre-caplacizumab era, mortality was higher than that reported in caplacizumab-treated patients in clinical trials conducted in highly experienced centers [16, 17]. However, these real-life data should be taken as less biased findings, and no data on post-marketing outcomes in TTP patients receiving caplacizumab have been published to date.

## Conclusions

In this large cohort of 1096 adults with TTP treated over a 10-year period in the pre-caplacizumab era, survival improved over time. Early TPE and rituximab were associated with improved survival suggesting their incorporation to the standard of care for TTP. Studies to evaluate emerging TTP therapies should add the drug to be tested to early TPE, first-line steroids, and rituximab. Control patients would need to be recruited synchronously, as survival improves over time.

## Abbreviations

TTP: thrombotic thrombocytopenic purpura
TPE: therapeutic plasma exchange
TMA: thrombotic microangiopathy
VWF: von Willebrand factor
ICD-9: International Classification of Diseases version 9
ICU: intensive care unit
